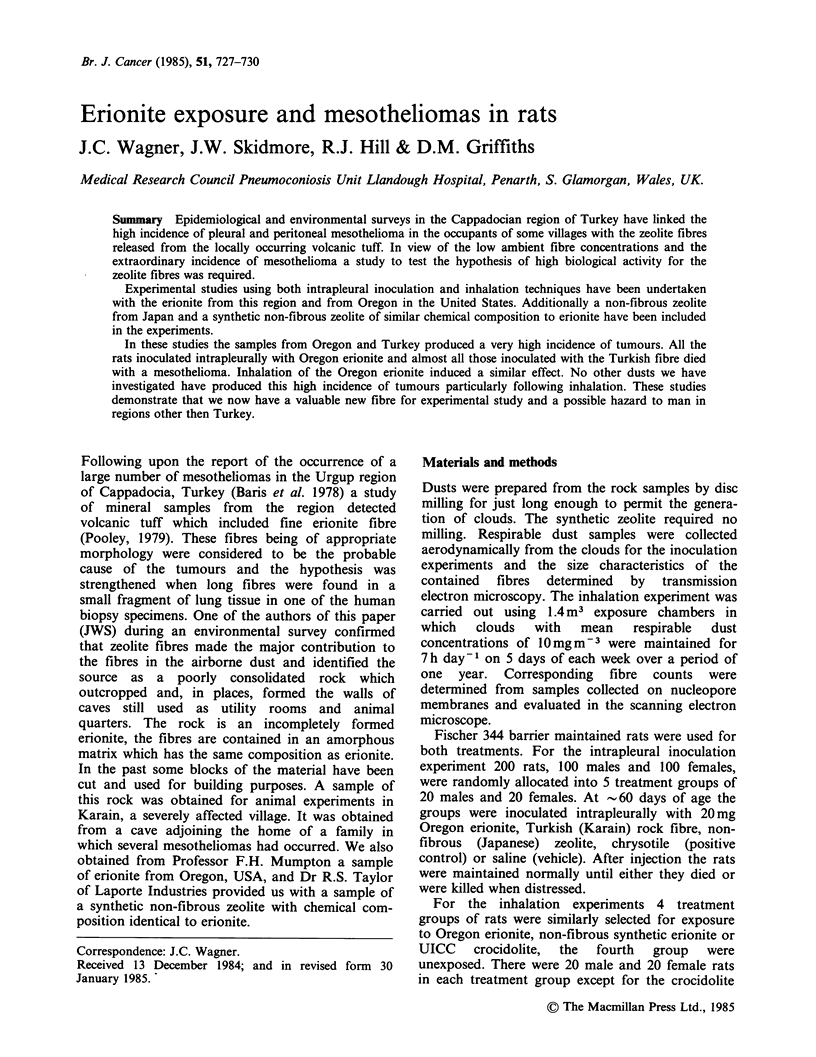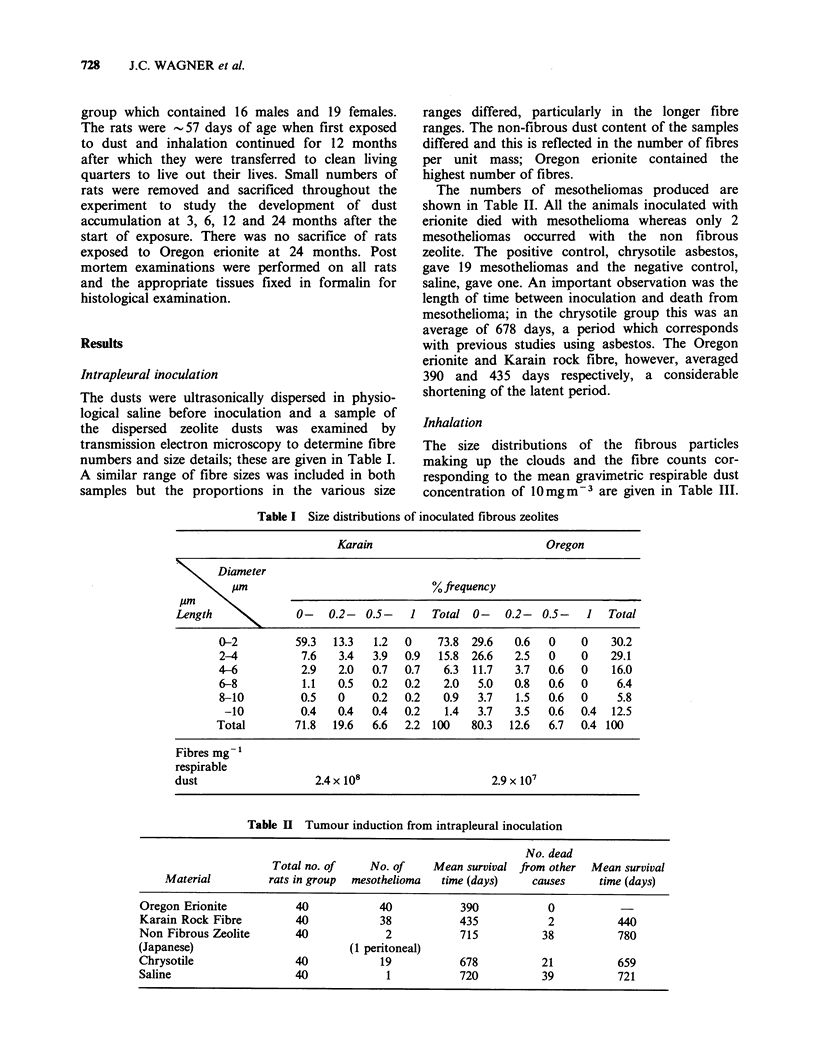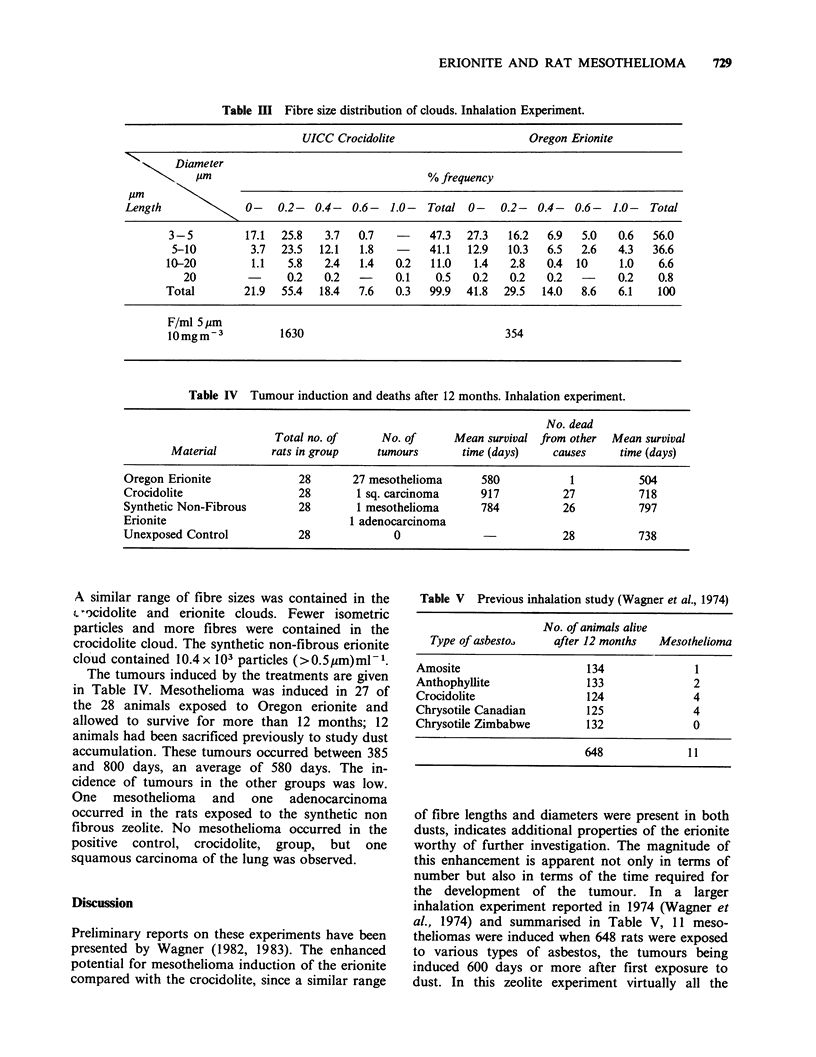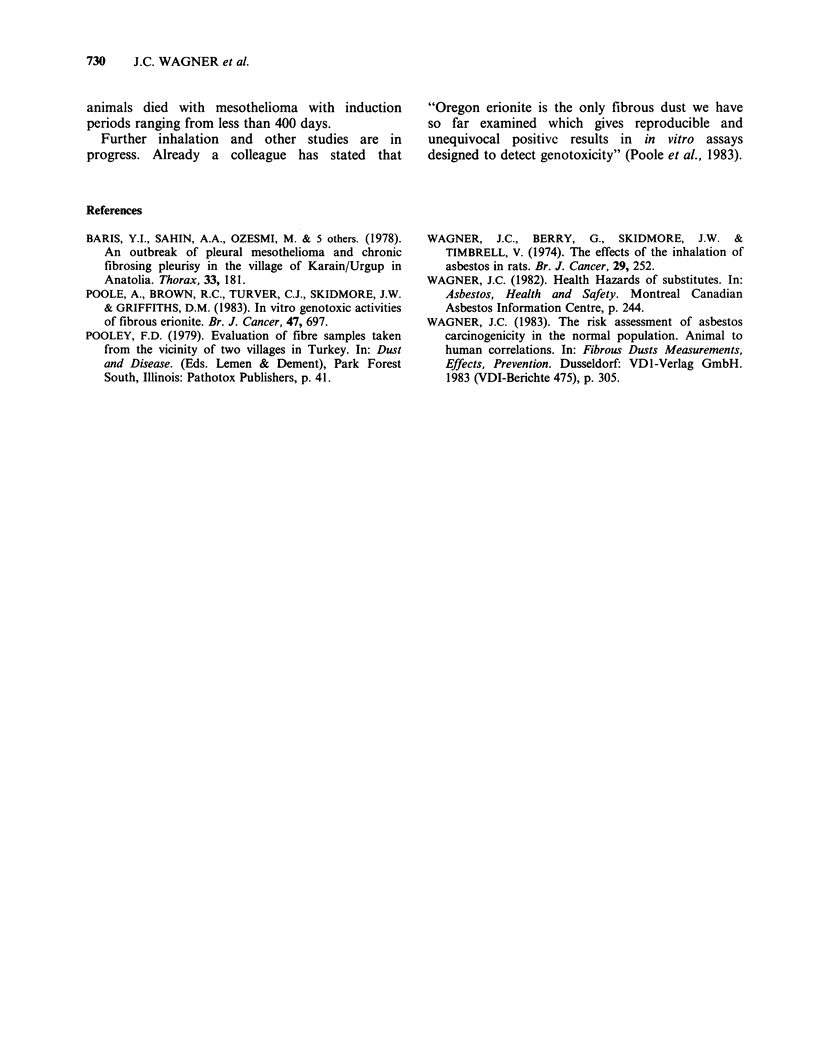# Erionite exposure and mesotheliomas in rats.

**DOI:** 10.1038/bjc.1985.108

**Published:** 1985-05

**Authors:** J. C. Wagner, J. W. Skidmore, R. J. Hill, D. M. Griffiths

## Abstract

Epidemiological and environmental surveys in the Cappadocian region of Turkey have linked the high incidence of pleural and peritoneal mesothelioma in the occupants of some villages with the zeolite fibres released from the locally occurring volcanic tuff. In view of the low ambient fibre concentrations and the extraordinary incidence of mesothelioma a study to test the hypothesis of high biological activity for the zeolite fibres was required. Experimental studies using both intrapleural inoculation and inhalation techniques have been undertaken with the erionite from this region and from Oregon in the United States. Additionally a non-fibrous zeolite from Japan and a synthetic non-fibrous zeolite of similar chemical composition to erionite have been included in the experiments. In these studies the samples from Oregon and Turkey produced a very high incidence of tumours. All the rats inoculated intrapleurally with Oregon erionite and almost all those inoculated with the Turkish fibre died with a mesothelioma. Inhalation of the Oregon erionite induced a similar effect. No other dusts we have investigated have produced this high incidence of tumours particularly following inhalation. These studies demonstrate that we now have a valuable new fibre for experimental study and a possible hazard to man in regions other then Turkey.


*Br. J. Cancer* (1985), **51**, 727–730

### Erionite exposure and mesotheliomas in rats

J.C. Wagner, J.W. Skidmore, R.J. Hill & D.M. Griffiths

*Medical Research Council Pneumoconiosis Unit Llandough Hospital, Penarth, S. Glamorgan, Wales, UK.*

**Summary** Epidemiological and environmental surveys in the Cappadocian region of Turkey have linked the high incidence of pleural and peritoneal mesothelioma in the occupants of some villages with the zeolite fibres released from the locally occurring volcanic tuff. In view of the low ambient fibre concentrations and the extraordinary incidence of mesothelioma a study to test the hypothesis of high biological activity for the zeolite fibres was required.

Experimental studies using both intrapleural inoculation and inhalation techniques have been undertaken with the erionite from this region and from Oregon in the United States. Additionally a non-fibrous zeolite from Japan and a synthetic non-fibrous zeolite of similar chemical composition to erionite have been included in the experiments.

In these studies the samples from Oregon and Turkey produced a very high incidence of tumours. All the rats inoculated intrapleurally with Oregon erionite and almost all those inoculated with the Turkish fibre died with a mesothelioma. Inhalation of the Oregon erionite induced a similar effect. No other dusts we have investigated have produced this high incidence of tumours particularly following inhalation. These studies demonstrate that we now have a valuable new fibre for experimental study and a possible hazard to man in regions other then Turkey.

Following upon the report of the occurrence of a large number of mesotheliomas in the Urgup region of Cappadocia, Turkey (Baris *et al.* 1978) a study of mineral samples from the region detected volcanic tuff which included fine erionite fibre (Pooley, 1979). These fibres being of appropriate morphology were considered to be the probable cause of the tumours and the hypothesis was strengthened when long fibres were found in a small fragment of lung tissue in one of the human biopsy specimens. One of the authors of this paper (JWS) during an environmental survey confirmed that zeolite fibres made the major contribution to the fibres in the airborne dust and identified the source as a poorly consolidated rock which outcropped and, in places, formed the walls of caves still used as utility rooms and animal quarters. The rock is an incompletely formed erionite, the fibres are contained in an amorphous matrix which has the same composition as erionite. In the past some blocks of the material have been cut and used for building purposes. A sample of this rock was obtained for animal experiments in Karain, a severely affected village. It was obtained from a cave adjoining the home of a family in which several mesotheliomas had occurred. We also obtained from Professor F.H. Mumpton a sample of erionite from Oregon, USA, and Dr R.S. Taylor of Laporte Industries provided us with a sample of a synthetic non-fibrous zeolite with chemical composition identical to erionite.

Correspondence: J.C. Wagner.
Received 13 December 1984; and in revised form 30 January 1985.

### Materials and methods

Dusts were prepared from the rock samples by disc milling for just long enough to permit the generation of clouds. The synthetic zeolite required no milling. Respirable dust samples were collected aerodynamically from the clouds for the inoculation experiments and the size characteristics of the contained fibres determined by transmission electron microscopy. The inhalation experiment was carried out using $1.4\,m^3$ exposure chambers in which clouds with mean respirable dust concentrations of $10\,mg\,m^{-3}$ were maintained for $7\,h\,day^{-1}$ on 5 days of each week over a period of one year. Corresponding fibre counts were determined from samples collected on nucleopore membranes and evaluated in the scanning electron microscope.

Fischer 344 barrier maintained rats were used for both treatments. For the intrapleural inoculation experiment 200 rats, 100 males and 100 females, were randomly allocated into 5 treatment groups of 20 males and 20 females. At ~60 days of age the groups were inoculated intrapleurally with 20 mg Oregon erionite, Turkish (Karain) rock fibre, non-fibrous (Japanese) zeolite, chrysotile (positive control) or saline (vehicle). After injection the rats were maintained normally until either they died or were killed when distressed.

For the inhalation experiments 4 treatment groups of rats were similarly selected for exposure to Oregon erionite, non-fibrous synthetic erionite or UICC crocidolite, the fourth group were unexposed. There were 20 male and 20 female rats in each treatment group except for the crocidolite

© The Macmillan Press Ltd., 1985

group which contained 16 males and 19 females. The rats were ~57 days of age when first exposed to dust and inhalation continued for 12 months after which they were transferred to clean living quarters to live out their lives. Small numbers of rats were removed and sacrificed throughout the experiment to study the development of dust accumulation at 3, 6, 12 and 24 months after the start of exposure. There was no sacrifice of rats exposed to Oregon erionite at 24 months. Post mortem examinations were performed on all rats and the appropriate tissues fixed in formalin for histological examination.

### Results

### *Intrapleural inoculation*

The dusts were ultrasonically dispersed in physiological saline before inoculation and a sample of the dispersed zeolite dusts was examined by transmission electron microscopy to determine fibre numbers and size details; these are given in Table I. A similar range of fibre sizes was included in both samples but the proportions in the various size ranges differed, particularly in the longer fibre ranges. The non-fibrous dust content of the samples differed and this is reflected in the number of fibres per unit mass; Oregon erionite contained the highest number of fibres.

The numbers of mesotheliomas produced are shown in Table II. All the animals inoculated with erionite died with mesothelioma whereas only 2 mesotheliomas occurred with the non fibrous zeolite. The positive control, chrysotile asbestos, gave 19 mesotheliomas and the negative control, saline, gave one. An important observation was the length of time between inoculation and death from mesothelioma; in the chrysotile group this was an average of 678 days, a period which corresponds with previous studies using asbestos. The Oregon erionite and Karain rock fibre, however, averaged 390 and 435 days respectively, a considerable shortening of the latent period.

### *Inhalation*

The size distributions of the fibrous particles making up the clouds and the fibre counts corresponding to the mean gravimetric respirable dust concentration of $10 \, mg \, m^{-3}$ are given in Table III.

**Table I** Size distributions of inoculated fibrous zeolites

| | Karain | | | | | Oregon | | | | |
|---|---|---|---|---|---|---|---|---|---|---|
| Diameter μm / Length μm | % frequency | | | | | | | | | |
| | 0– | 0.2– | 0.5– | 1 | Total | 0– | 0.2– | 0.5– | 1 | Total |
| 0–2 | 59.3 | 13.3 | 1.2 | 0 | 73.8 | 29.6 | 0.6 | 0 | 0 | 30.2 |
| 2–4 | 7.6 | 3.4 | 3.9 | 0.9 | 15.8 | 26.6 | 2.5 | 0 | 0 | 29.1 |
| 4–6 | 2.9 | 2.0 | 0.7 | 0.7 | 6.3 | 11.7 | 3.7 | 0.6 | 0 | 16.0 |
| 6–8 | 1.1 | 0.5 | 0.2 | 0.2 | 2.0 | 5.0 | 0.8 | 0.6 | 0 | 6.4 |
| 8–10 | 0.5 | 0 | 0.2 | 0.2 | 0.9 | 3.7 | 1.5 | 0.6 | 0 | 5.8 |
| –10 | 0.4 | 0.4 | 0.4 | 0.2 | 1.4 | 3.7 | 3.5 | 0.6 | 0.4 | 12.5 |
| Total | 71.8 | 19.6 | 6.6 | 2.2 | 100 | 80.3 | 12.6 | 6.7 | 0.4 | 100 |
| Fibres $mg^{-1}$ respirable dust | $2.4 \times 10^8$ | | | | | $2.9 \times 10^7$ | | | | |

**Table II** Tumour induction from intrapleural inoculation

| *Material* | *Total no. of rats in group* | *No. of mesothelioma* | *Mean survival time (days)* | *No. dead from other causes* | *Mean survival time (days)* |
|---|---|---|---|---|---|
| Oregon Erionite | 40 | 40 | 390 | 0 | — |
| Karain Rock Fibre | 40 | 38 | 435 | 2 | 440 |
| Non Fibrous Zeolite (Japanese) | 40 | 2 (1 peritoneal) | 715 | 38 | 780 |
| Chrysotile | 40 | 19 | 678 | 21 | 659 |
| Saline | 40 | 1 | 720 | 39 | 721 |

**Table III** Fibre size distribution of clouds. Inhalation Experiment.

| *μm Length* \ *Diameter μm* | *UICC Crocidolite* | | | | | | *Oregon Erionite* | | | | | |
|---|---|---|---|---|---|---|---|---|---|---|---|---|
| | *% frequency* | | | | | | | | | | | |
| | *0–* | *0.2–* | *0.4–* | *0.6–* | *1.0–* | *Total* | *0–* | *0.2–* | *0.4–* | *0.6–* | *1.0–* | *Total* |
| 3–5 | 17.1 | 25.8 | 3.7 | 0.7 | — | 47.3 | 27.3 | 16.2 | 6.9 | 5.0 | 0.6 | 56.0 |
| 5–10 | 3.7 | 23.5 | 12.1 | 1.8 | — | 41.1 | 12.9 | 10.3 | 6.5 | 2.6 | 4.3 | 36.6 |
| 10–20 | 1.1 | 5.8 | 2.4 | 1.4 | 0.2 | 11.0 | 1.4 | 2.8 | 0.4 | 10 | 1.0 | 6.6 |
| 20 | — | 0.2 | 0.2 | — | 0.1 | 0.5 | 0.2 | 0.2 | 0.2 | — | 0.2 | 0.8 |
| Total | 21.9 | 55.4 | 18.4 | 7.6 | 0.3 | 99.9 | 41.8 | 29.5 | 14.0 | 8.6 | 6.1 | 100 |
| F/ml 5 μm $10\,mg\,m^{-3}$ | 1630 | | | | | | 354 | | | | | |

**Table IV** Tumour induction and deaths after 12 months. Inhalation experiment.

| *Material* | *Total no. of rats in group* | *No. of tumours* | *Mean survival time (days)* | *No. dead from other causes* | *Mean survival time (days)* |
|---|---|---|---|---|---|
| Oregon Erionite | 28 | 27 mesothelioma | 580 | 1 | 504 |
| Crocidolite | 28 | 1 sq. carcinoma | 917 | 27 | 718 |
| Synthetic Non-Fibrous Erionite | 28 | 1 mesothelioma<br>1 adenocarcinoma | 784 | 26 | 797 |
| Unexposed Control | 28 | 0 | — | 28 | 738 |

A similar range of fibre sizes was contained in the crocidolite and erionite clouds. Fewer isometric particles and more fibres were contained in the crocidolite cloud. The synthetic non-fibrous erionite cloud contained $10.4 \times 10^3$ particles ($>0.5\,\mu m$)$ml^{-1}$.

The tumours induced by the treatments are given in Table IV. Mesothelioma was induced in 27 of the 28 animals exposed to Oregon erionite and allowed to survive for more than 12 months; 12 animals had been sacrificed previously to study dust accumulation. These tumours occurred between 385 and 800 days, an average of 580 days. The incidence of tumours in the other groups was low. One mesothelioma and one adenocarcinoma occurred in the rats exposed to the synthetic non fibrous zeolite. No mesothelioma occurred in the positive control, crocidolite, group, but one squamous carcinoma of the lung was observed.

### Discussion

Preliminary reports on these experiments have been presented by Wagner (1982, 1983). The enhanced potential for mesothelioma induction of the erionite compared with the crocidolite, since a similar range

**Table V** Previous inhalation study (Wagner *et al.*, 1974)

| *Type of asbestos* | *No. of animals alive after 12 months* | *Mesothelioma* |
|---|---|---|
| Amosite | 134 | 1 |
| Anthophyllite | 133 | 2 |
| Crocidolite | 124 | 4 |
| Chrysotile Canadian | 125 | 4 |
| Chrysotile Zimbabwe | 132 | 0 |
| | 648 | 11 |

of fibre lengths and diameters were present in both dusts, indicates additional properties of the erionite worthy of further investigation. The magnitude of this enhancement is apparent not only in terms of number but also in terms of the time required for the development of the tumour. In a larger inhalation experiment reported in 1974 (Wagner *et al.*, 1974) and summarised in Table V, 11 mesotheliomas were induced when 648 rats were exposed to various types of asbestos, the tumours being induced 600 days or more after first exposure to dust. In this zeolite experiment virtually all the animals died with mesothelioma with induction periods ranging from less than 400 days.

Further inhalation and other studies are in progress. Already a colleague has stated that "Oregon erionite is the only fibrous dust we have so far examined which gives reproducible and unequivocal positivc results in *in vitro* assays designed to detect genotoxicity" (Poole *et al.*, 1983).

### References

BARIS, Y.I., SAHIN, A.A., OZESMI, M. & 5 others. (1978). An outbreak of pleural mesothelioma and chronic fibrosing pleurisy in the village of Karain/Urgup in Anatolia. *Thorax*, **33**, 181.

POOLE, A., BROWN, R.C., TURVER, C.J., SKIDMORE, J.W. & GRIFFITHS, D.M. (1983). In vitro genotoxic activities of fibrous erionite. *Br. J. Cancer*, **47**, 697.

POOLEY, F.D. (1979). Evaluation of fibre samples taken from the vicinity of two villages in Turkey. In: *Dust and Disease*. (Eds. Lemen & Dement), Park Forest South, Illinois: Pathotox Publishers, p. 41.

WAGNER, J.C., BERRY, G., SKIDMORE, J.W. & TIMBRELL, V. (1974). The effects of the inhalation of asbestos in rats. *Br. J. Cancer*, **29**, 252.

WAGNER, J.C. (1982). Health Hazards of substitutes. In: *Asbestos, Health and Safety*. Montreal Canadian Asbestos Information Centre, p. 244.

WAGNER, J.C. (1983). The risk assessment of asbestos carcinogenicity in the normal population. Animal to human correlations. In: *Fibrous Dusts Measurements, Effects, Prevention*. Dusseldorf: VD1-Verlag GmbH. 1983 (VDI-Berichte 475), p. 305.